# Inactivation of the transforming growth factor **β** type II receptor in human small cell lung cancer cell lines

**DOI:** 10.1038/sj.bjc.6690161

**Published:** 1999-03

**Authors:** S Hougaard, P Nørgaard, N Abrahamsen, H L Moses, M Spang-Thomsen, H Skovgaard Poulsen

**Affiliations:** Section for Radiation Biology, The Finsen Center, University Hospital of Copenhagen, Copenhagen, Denmark; Institute of Molecular Pathology, Copenhagen University, Copenhagen, Denmark; Vanderbilt Cancer Center, Vanderbilt University, Nashville, Tennessee, USA

**Keywords:** small cell lung cancer, cell lines, transforming growth factor β, type II receptor, mutation, transcription

## Abstract

Transforming growth factor β (TGF-β) exerts a growth inhibitory effect on many cell types through binding to two types of receptors, the type I and II receptors. Resistance to TGF-β due to lack of type II receptor (RII) has been described in some cancer types including small cell lung cancer (SCLC). The purpose of this study was to examine the cause of absent RII expression in SCLC cell lines. Northern blot analysis showed that RII RNA expression was very weak in 16 of 21 cell lines. To investigate if the absence of RII transcript was due to mutations, we screened the poly-A tract for mutations, but no mutations were detected. Additional screening for mutations of the RII gene revealed a GG to TT base substitution in one cell line, which did not express RII. This mutation generates a stop codon resulting in predicted synthesis of a truncated RII of 219 amino acids. The nature of the mutation, which has not previously been observed in RII, has been linked to exposure to benzo[a]-pyrene, a component of cigarette smoke. Since RII has been mapped to chromosome 3p22 and nearby loci are often hypermethylated in SCLC, it was examined whether the lack of RII expression was due to hypermethylation. Southern blot analysis of the RII promoter did not show altered methylation patterns. The restriction endonuclease pattern of the RII gene was altered in two SCLC cell lines when digested with *Sma* 1. However, treatment with 5-aza-2′-deoxycytidine did not induce expression of RII mRNA. Our results indicate that in SCLC lack of RII mRNA is not commonly due to mutations and inactivation of RII transcription was not due to hypermethylation of the RII promoter or gene. Thus, these data show that in most cases of the SCLC cell lines, the RII gene and promoter is intact in spite of absent RII expression. However, the nature of the mutation found could suggest that it was caused by cigarette smoking. © 1999 Cancer Research Campaign


*British Journal of Cancer* (1999) **79**(7/8), 1005–1011
© 1999 Cancer Research Campaign
Article no. bjoc.1998.0161

### Inactivation of the transforming growth factor β type II receptor in human small cell lung cancer cell lines

**S Hougaard[1], P Nørgaard[1], N Abrahamsen[1], HL Moses[3], M Spang-Thomsen[2] and H Skovgaard Poulsen[1]**

[1]Section for Radiation Biology, The Finsen Center, University Hospital of Copenhagen, Copenhagen, Denmark; [2]Institute of Molecular Pathology, Copenhagen University, Copenhagen, Denmark; [3]Vanderbilt Cancer Center, Vanderbilt University, Nashville, Tennessee, USA

**Summary** Transforming growth factor β (TGF-β) exerts a growth inhibitory effect on many cell types through binding to two types of receptors, the type I and II receptors. Resistance to TGF-β due to lack of type II receptor (RII) has been described in some cancer types including small cell lung cancer (SCLC). The purpose of this study was to examine the cause of absent RII expression in SCLC cell lines. Northern blot analysis showed that RII RNA expression was very weak in 16 of 21 cell lines. To investigate if the absence of RII transcript was due to mutations, we screened the poly-A tract for mutations, but no mutations were detected. Additional screening for mutations of the RII gene revealed a GG to TT base substitution in one cell line, which did not express RII. This mutation generates a stop codon resulting in predicted synthesis of a truncated RII of 219 amino acids. The nature of the mutation, which has not previously been observed in RII, has been linked to exposure to benzo[a]-pyrene, a component of cigarette smoke. Since RII has been mapped to chromosome 3p22 and nearby loci are often hypermethylated in SCLC, it was examined whether the lack of RII expression was due to hypermethylation. Southern blot analysis of the RII promoter did not show altered methylation patterns. The restriction endonuclease pattern of the RII gene was altered in two SCLC cell lines when digested with *Sma*1. However, treatment with 5-aza-2′-deoxycytidine did not induce expression of RII mRNA. Our results indicate that in SCLC lack of RII mRNA is not commonly due to mutations and inactivation of RII transcription was not due to hypermethylation of the RII promoter or gene. Thus, these data show that in most cases of the SCLC cell lines, the RII gene and promoter is intact in spite of absent RII expression. However, the nature of the mutation found could suggest that it was caused by cigarette smoking.

**Keywords:** small cell lung cancer; cell lines; transforming growth factor β; type II receptor; mutation; transcription

Transforming growth factor β (TGF-β) is an important regulator of cell proliferation, differentiation and formation of extracellular matrix (Roberts and Sporn, 1990; Moses, 1992; Kingsley, 1994). The effects of TGF-β are mediated through binding to two ubiquitously expressed receptors, TGF-β receptor type I (RI), and type II (RII). RI and RII contain cytoplasmic serine/threonine kinase domains and act in concert by heterodimerization (Wrana et al, 1994). One of the most pronounced effects of TGF-β in vitro is growth inhibition of ectodermally derived cells (for review see Nørgaard et al, 1995). Some carcinomas are resistant to the growth inhibitory effect of TGF-β, which in several cases has been ascribed to lack of RII (Geiser et al, 1992; Inagaki et al, 1993; Nørgaard et al, 1994; Park et al, 1994). Recently, it was shown that TGF-$\beta_1$ acts as a growth inhibitor of carcinomas in vivo, at least during early tumour growth (Cui et al, 1994, 1996; Pierce et al, 1995). In colon and gastric cancer, showing the replication error phenotype, a high incidence of mutations in the poly-adenosine (poly-A) tract of the RII gene was found leading to loss of RII (Markowitz et al, 1995).

Lung cancer is divided into small cell lung cancer (SCLC) and non-small cell lung cancer (NSCLC). Both types are characterized by gross chromosomal rearrangements of which loss of heterozygosity at the short arm of chromosome 3 is very frequent (Mitsudomi et al, 1996; Todd et al, 1997). Interestingly, the RII gene has been reported to map to chromosome 3p22 (Mathew et al, 1994) and nearby loci have been found to be hypermethylated in SCLC (Makos et al, 1995). Of 35 SCLC and NSCLC tumour specimens with chromosome 3p loss examined for RII mutations, only one SCLC tumour harboured a mutation in the poly-A tract. The SCLC tumour showed a replication error phenotype (Tani et al, 1997). Mutations in the poly-A tract of the RII gene therefore seem to be infrequent in lung cancer.

We have previously shown that a number of SCLC cell lines did not express RII protein (Damstrup et al, 1993), rendering them resistant to the growth inhibitory effect of TGF$\beta_1$ (Nørgaard et al, 1994, 1996). In this report we characterized the expression of RNA for RII in a panel of 21 SCLC cell lines and found that 16 cell lines showed very weak expression. In one cell line we identified a mutation generating a stop codon which predicts synthesis of a truncated RII protein lacking the serine/threonine kinase domain. This particular type of mutation has been linked to exposure to benzo[a]-pyrene, a component of cigarette smoke. We investigated whether lack of RII transcript was due to hypermethylation of the promoter, but found that this was not the case. In two cell lines Southern blot analysis revealed an altered restriction pattern of the RII gene, but treatment with 5-aza-2′-deoxycytidine (5-azaCdR) did not increase RII expression. We conclude that in the majority of SCLC cell lines, the RII gene and promoter is intact. However, interestingly, the nature of the found mutation suggests that it could be attributed to cigarette smoking.

*Received 30 January 1998*
*Revised 21 July 1998*
*Accepted 18 August 1998*

*Correspondence to:* H Skovgaard Poulsen, Section for Radiation Biology, The Finsen Center, University Hospital of Copenhagen, Blegdamsvej 9, DK-2100 Copenhagen, Denmark

### MATERIALS AND METHODS

### Cell lines

SCLC cell lines were cultured in 150 cm$^2$ culture flasks at 37°C under standard conditions in medium containing 10% heat inactivated fetal calf serum (FCS; Gibco BRL) without antibiotics. All cell lines were routinely tested for mycoplasma contamination and were found negative. Eight cell lines (DMS) were established at Dartmouth Medical School (Hanover, NH, USA) and cultured in Waymouth medium (GIBCO). Seven cell lines (GLC) were established at Groningen Lung Cancer Centre (Groningen, The Netherlands), two cell lines (NCI) were established at the National Cancer Institute (Bethesda, MD, USA), and two cell lines (24H and 86M1) were established in Marburg, Germany. They were cultured in RPMI-1640 (GIBCO). Finally, two cell lines (CPH) were established in our own laboratory (Copenhagen, Denmark) and cultured in Eagle's minimum essential medium (EMEM; GIBCO). The origin and establishment of the cell lines have previously been described in (Pettengill et al, 1980; Carney et al, 1985; De Leij et al, 1985; Engelholm et al, 1986; Berendsen et al, 1988; Vincent et al, 1997). Primary human foreskin fibroblasts (HFF; a gift from O Pettengill, Dartmouth Medical School) were grown in Hanks basal medium Eagle (BME; GIBCO). Cells for investigations were harvested during exponential growth, washed in phosphate-buffered saline (PBS) (Dulbeccos; GIBCO), centrifuged at 300 ***g***, immediately frozen in liquid nitrogen, and stored at – 80°C.

### RNA extraction, electrophoresis and blotting

Total RNA was isolated by use of TRIzol$^8$LS REagent (GibcoBRL, Life Technologies) as recommended. Subsequent Northern blot analysis was performed as previously described (Nørgaard et al, 1995). The Northern blots were analysed by phosphor imager (STORM, Molecular Dynamics). The blots were hybridized with a $^{32}$P-labelled RII probe, a 930 bp *Pst*I–*Sac*I fragment of the cloned 4.5 kb H2-3FF human cDNA, kindly provided by HY Lin (Lin et al, 1992). To ensure equal loading of RNA present on the membrane, the blots were hybridized with a $^{32}$P-labelled glyceraldehyde 3-phosphate dehydrogenase (GAPDH) probe, a 1.1 kb cDNA fragment purchased from Clontech hybridizing to a single ~ 1.4 kb mRNA.

### SSCP analysis

From each cell line, cDNA was obtained by reverse transcriptase polymerase chain reaction (RT-PCR) according to recommendations (1st Strand cDNA Synthesis Kit for RT-PCR, Boehringer Mannheim). A 269 basepair (bp) GAPDH fragment was generated by PCR to test the cDNA reaction by use of the primers described in Liabakk et al (1996). A 1678 bp RII fragment (from bp 35 to 1713) was generated by PCR with the primers: 5′-GCACATCGTCCT-GTGGACGCGTA and 5′-CCTGCCCCATAAAGAGCTATTTGG.

For subsequent generation of 250 to 363 bp fragments the following primers were used:

From bp – 304 to 59: 5′-GCGCTGAGTTGAAGTTGAGTGAGT
5′-ATACGCGTCCACAGGAACGATGTGC.

In this case single-stranded confirmational polymorphism (SSCP) was performed on PCR fragments generated utilizing genomic DNA as template.

| | |
|---|---|
| From bp 35 to 310: | 5′-GCACATCCTGTGGACGCGTA<br>5′-GGTCATGGGAAACTGTCTCT. |
| From bp 242 to 543: | 5′-GGAAGTCTGTGTGTGGCTG-TATGG<br>5′-CAGATATGGCAACTCCCAGTGG. |
| From bp 491 to 780: | 5′-ATAAGTGACAGGCATCAGC<br>5′-CCTTATAGACCTCAGCAAAGCG. |
| From bp 705 to 1011: | 5′-AACCACAACACAGAGCTGCTGC<br>5′-TACTCCTGTAGGTTGCCCTTGC. |
| From bp 943 to 1193: | 5′-CGGAGTTGGGGAAACAATACTGG<br>5′-AAAGTCACAC-AGGCAGCAGG. |
| From bp 1149 to 1405: | 5′-AATATCCTCGTGAAGAACGACC<br>5′-TCTTTTACTTCTCCCACTGC |
| From bp 1356 to 1683: | 5′-GTGCTCTGGGAAATG-ACATCTCG<br>5′-AGCCGTCTTCAGGAATCTTCTCC. |

PCR comprised 30 cycles at 94°C for 30 s, 55–60°C for 1 min, and 72°C for 1 min to generate the 250–327 bp fragments and 2 min for the 1678 bp fragment. The PCR reactions were carried out with Thermoprime Plus DNA polymerase (Applied Biochemistry) in standard solutions. SSCP analysis was performed using non-denaturating 5% acrylamide gels with 0%, 5%, or 10% glycerol. The running conditions were 400 V for 1.5–2.5 h at 5°C or 10°C using a vertical electrophoresis apparatus (Hoefer, serie 600). The gels were stained with SYBR green (Medinova) and analysed by a fluorescence imager (STORM, Molecular Dynamics).

### DNA sequencing

DNA fragments, which showed aberrant migration in the SSCP analysis were de novo amplified by PCR. PCR products were purified using MicroSpin S-400 HR Columns (Pharmacia). The fragments were sequenced by Thermo Sequenase $^{33}$P-labelled terminator cycle sequencing kit (Amersham, Life Science). The samples were run on a standard acrylamide/urea gel and autoradiography was subsequently performed.

### Analysis of the poly-A tract

A fragment from bp 329 to 401 from each cell line was generated by PCR as previously described (Myeroff et al, 1995) using genomic DNA as template. The samples were electrophoresed on a 6% acrylamide gel followed by autoradiography.

### DNA extraction, electrophoresis and blotting

DNA was extracted with phenol and chloroform by standard methods (Sambrook et al, 1989), and digested with the restriction endonucleases (RE): *Bsm*I with one of the following methylation sensitive RE: *Sma*I, *Bst*4I, *Hin*PI, *Aat*II, each of which has one recognition site in the 3′-end of the promoter and the 5′-end of the RII untranslated region. Digestion was performed as recommended by the supplier (New England Biolabs) using 10 U of each methylation sensitive RE/mg DNA and incubation for 18 h. DNA (10 mg/lane) was electrophoresed on 0.8% agarose gels and transferred to charged nylon membranes (GeneScreen*Plus*, NEN/DuPont). Prehybridization, hybridization and washing were performed as recommended by the supplier. Washing stringency was 2 × SSC, 1% sodium dodecyl sulphate twice for 30 min at 60°C. Southern blots were probed with both $^{32}$P-labelled full-length RII cDNA probe and a probe for the promoter. The latter

 © Cancer Research Campaign 1999

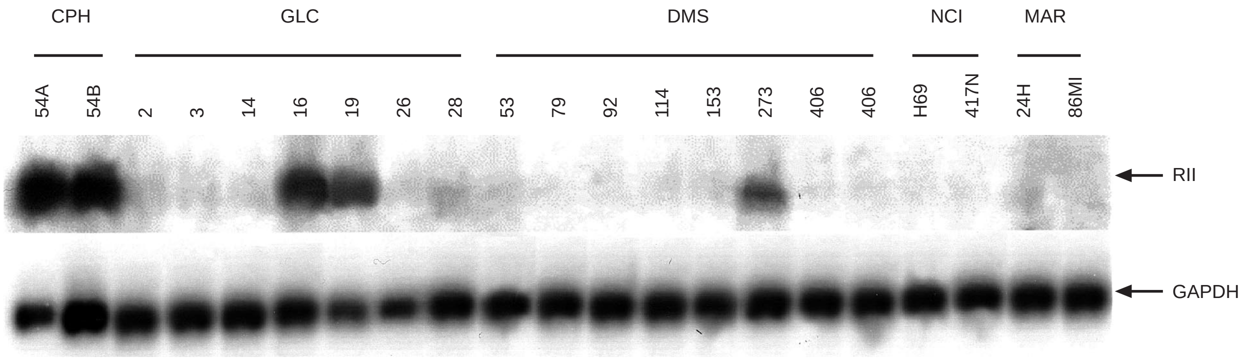

**Figure 1** Northern blot analysis of RII RNA in 21 SCLC cell lines. Total RNA (10 µg/lane) were electrophoresed in formaldehyde gel, transferred to nylon membranes and subsequently hybridized with the human RII probe. The membrane was reprobed with GAPDH as internal standard. The cell lines CPH 54A, CPH 54B, GLC 16, GLC 19 and DMS 273 express RII RNA, while the rest of the cell lines express RII RNA very weakly probe was generated by PCR using the primers 5′-ATCTG-GTTGCCCTAGCAAGA and 5′-ATACGCGTCCACAGGAAC-GATGTGC, which generated a fragment of 1306 bp covering the region from nucleotide –1247 to 59. The template was genomic DNA from HFF. Membranes were exposed to X-ray film (Amersham) at – 80°C with an intensifying screen for 7 days.

### Treatment with 5-azaCdR

Two cell lines showing altered RE-pattern with digestion with *Sma*I, DMS 79, DMS 114 (both RII-negative), GLC 2 (RII-negative), and CPH 54A (RII-positive) were treated with 0.5, 0.75 and 2.0 mM 5-azaCdR (Sigma). The cells were treated for two doubling times (respectively 83, 31, 26 and 43 h for each cell line). The cells were harvested as described above at start, at one doubling time and at two doubling times. To demonstrate a possible induction of RII, total RNA was isolated by use of TRIzol®LS REagent (GibcoBRL, Life Technologies) as recommended. cDNA was obtained as described in SSCP analysis, and expression of the receptor was detected by use of the above mentioned primers generating a 508 bp 5′-end transcript.

### RESULTS

### RII mRNA expression

Expression of RII mRNA was examined in 21 SCLC cell lines by Northern blot analysis. The cell lines CPH 54A, CPH 54B, GLC 16, GLC 19 and DMS 273 expressed significant amounts of a ~5.5 kb mRNA for RII, whereas the rest of the cell lines showed very weak expression (Figure 1). This correlated with previous findings where RII protein expression was detected by chemical cross-linking (Damstrup et al, 1993).

### Screening for mutations of RII

It has previously been reported that mutations in the poly-A tract of the RII gene have been associated with reduced, or lack of, RII transcript (Markowitz et al, 1995) and hence we analysed the cell lines for mutations in the poly-A tract (nucleotides 383–392) but found no alterations (data not shown). Next we screened the RII gene for mutation by SSCP analysis followed by sequencing. In two cell lines, GLC 26 and DMS 456, the fragment representing bp 491–780 migrated aberrantly (Figure 2). Subsequent sequencing revealed a mutation in DMS 456. The mutation was a GG to TT base substitution at nucleotides 658–659 (Figure 3A). This substitution converts the wild-type glutamic acid to a stop codon at amino acid 220 in exon 4 (Figure 3B). The presence of the mutation was confirmed at the level of genomic DNA. The transcript mutated in the serine/threonine kinase domain encodes a predicted inactive RII receptor if translated. The aberrant migrating band in GLC 26 was of lower molecular weight than in case of DMS 456 (Figure 2). The band was reproduced at several occasions, but it is presumably of no significance, since sequencing did not reveal any mutation in GLC 26.

### Analysis for altered methylation pattern

To investigate the reason for lack of RII transcript in 16 of the 21 SCLC cell lines, we examined all the SCLC cell lines for altered methylation pattern in the 3′-end of the promoter and the 5′-end of the untranslated region of the RII gene. This region fulfills the criteria for a C/G island, having a C/G content over 70%. Digestion with four different methylation sensitive REs, each having recognition sites in the RII promoter, followed by Southern blotting and hybridization with RII promoter probe, did not reveal alterations of the promoter and with that no signs of altered methylation of the RII promoter.

However, when the same Southern blots were hybridized with the probe for the RII gene, it was found that in the case of the *Sma*I/*Bsm*I digestion the RE pattern was altered in two cell lines, DMS 79 and DMS 114 (Figure 4). Digestion with the three other combinations of REs showed an identical pattern in all the SCLC cell lines indicating no gross structural changes of the RII gene (data not shown). To examine if the altered RE pattern in DMS 79 and DMS 114 could be due to hypermethylation of the RII gene, which could inactivate RII expression, we treated the cell lines with 5-azaCdR. The response to 5-azaCdR treatment was evaluated by detecting increased induction of RII RNA transcript. Treatment with 5-azaCdR for two doubling times did not increase transcription of the RII gene in either the two cell lines, or in another RII-negative cell line (GLC 2), and transcription did not increase in a RII-positive cell line (CPH 54A). We therefore concluded that the silencing of the RII gene was not due to hypermethylation of the RII gene in DMS 79 and DMS 114. The observation that the two cell lines exhibited an altered RE pattern with

© Cancer Research Campaign 1999

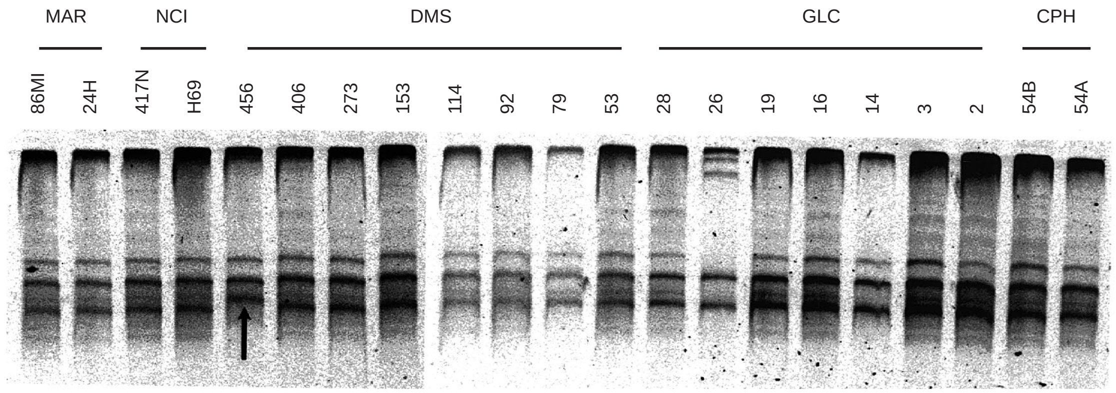

**Figure 2** SSCP gel run with the fragment from bp 491–780 of TGF-β–RII. Two RII-negative cell lines, GLC 26 and DMS 456, showed aberrantly migrating fragments. Only in DMS 456, indicated by arrow, the fragment contained a mutation

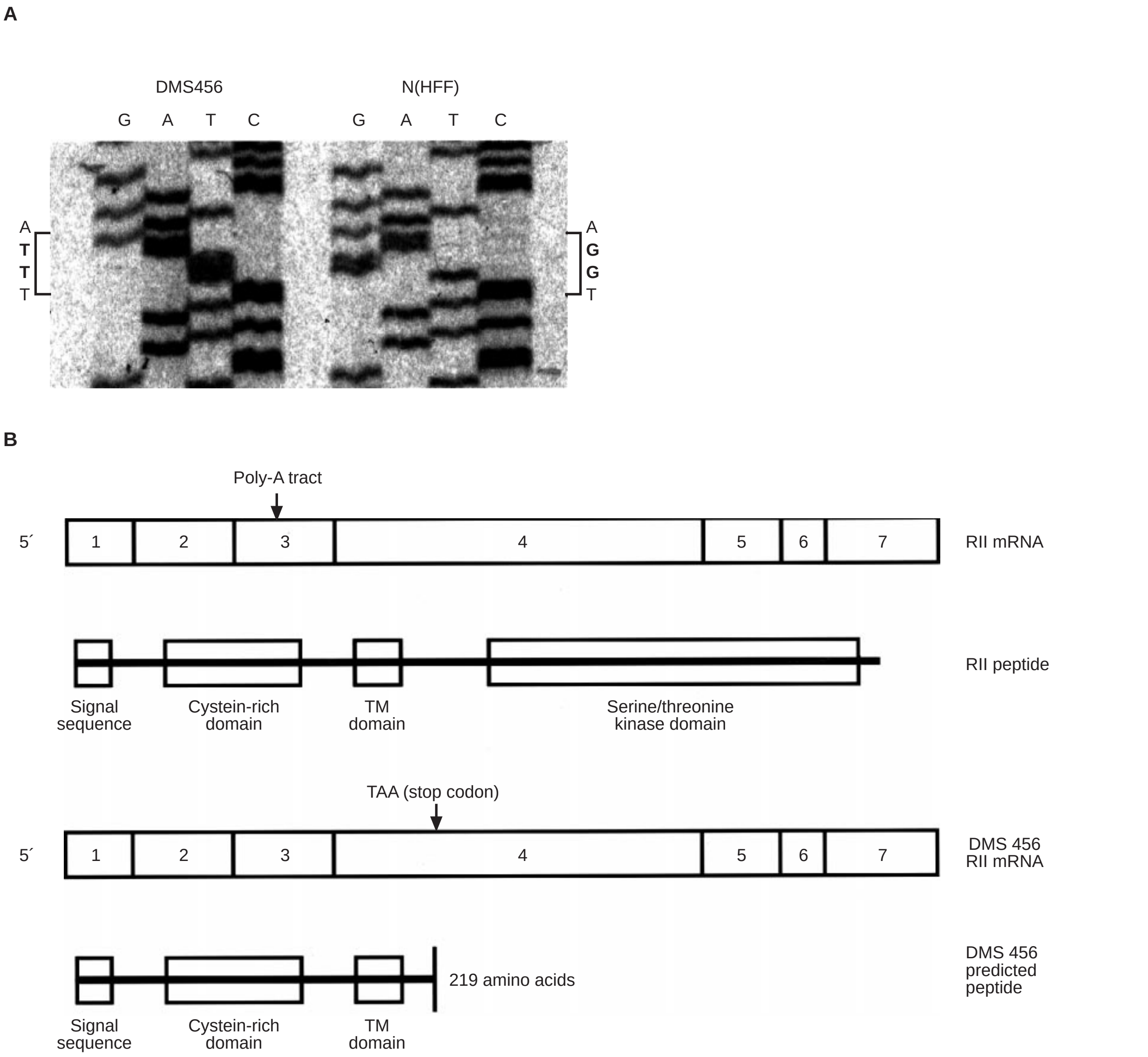

**Figure 3** Mutations of the RII gene detected by DNA sequencing. (**A**) DNA fragments showing different mobility were re-amplified by PCR using the corresponding set of primers and sequenced. In the SCLC cell line DMS 456 there was a GG→TT substitution in exon 4 at nucleotide 658–659. The corresponding wild-type sequence is displayed in line N. The region containing the mutations are marked in brackets and the mutations are indicated in bold. (**B**) Schematic representations of the sequence of wild-type RII mRNA and RII mRNA from DMS 456. The seven exons are indicated by numbers and the predicted translated protein is illustrated with the signal sequence, the cysteine-rich sequence, the transmembrane (TM) and the serine/threonine kinase domain

 *© Cancer Research Campaign 1999*

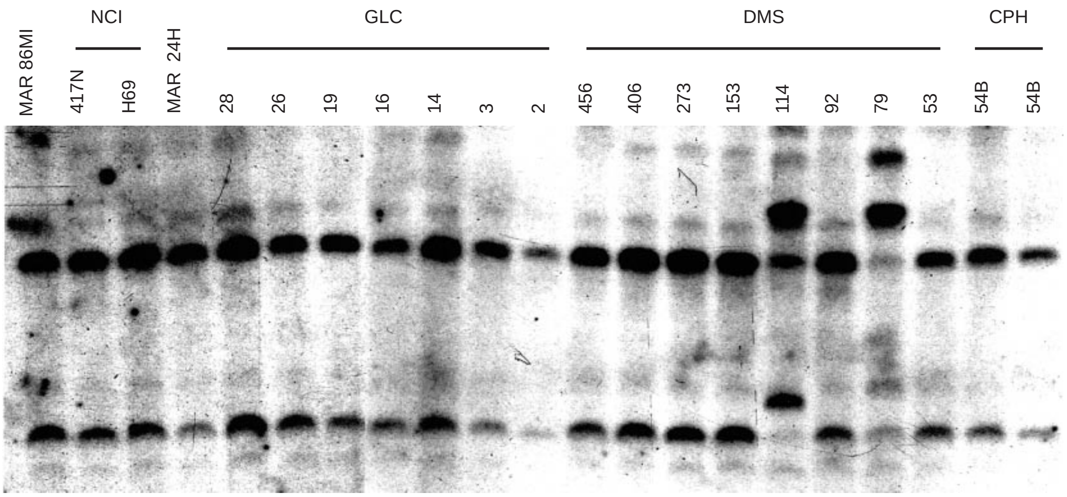

**Figure 4** Southern blot performed with genomic DNA from 21 SCLC cell lines digested with *Bsm*I and the methylation sensitive *Sma*I. The RII-negative cell lines DMS 79 and DMS 114 showed an altered digestion pattern digestion with *Sma*I could indicate that they contain an altered sequence in an intron at a particular recognition site for *Sma*I. Although the genomic structure of the RII gene has recently been described (Lu et al, 1996), the sequence of the introns between the seven exons has not been described. Accordingly, the precise restriction pattern cannot be deduced.

### DISCUSSION

We previously found that a number of SCLC cell lines did not express RII protein (Damstrup et al, 1993) and that sensitivity to the growth inhibitory effect of TGF$\beta_1$ correlated to expression of RII in five cell lines, while four cell lines not expressing RII were resistant to TGF$\beta_1$ (Nørgaard et al, 1994, 1996). We examined 21 SCLC cell lines for expression of RII RNA. Sixteen of the 21 cell lines showed weak expression of the RII RNA (Figure 1), which correlated with the previously published protein expression (Damstrup et al, 1993).

Mutations in the RII gene have been detected in human cancers of the colon (Lu et al, 1995; Markowitz et al, Myeroff et al, 1995), stomach (Park et al, 1994), endometrium (Myeroff et al, 1995), and head and neck (Garrigue-Antar et al, 1995). These observations are consistent with the proposal that RII is a tumour suppressor gene and that inactivation of RII could be involved in human carcinogenesis (Sun et al, 1994; Wang et al, 1995). RII mutations have been detected frequently in the poly-A tract in tumours with microsatellite instability (Lu et al, 1995; Markowitz et al, 1995; Myeroff et al, 1995), suggesting that RII mutations occur preferentially in microsatellite regions and in tumours with genomic instability. We did not find mutations in the poly-A tract, which is in agreement with another study where only one of 15 SCLC tumour specimens harboured a mutation at this site (Tani et al, 1997). Mutations in the serine/threonine kinase domain of the RII gene have also been found (Lu et al, 1995; Garrigue-Antar et al, 1997) and in this paper we describe a mutation in exon 4, which results in a truncated receptor lacking the kinase domain. However, this mutation per se cannot account for the observed lack of RII RNA. Recently, it has been described that 11 SCLC cell lines expressed low levels of RII, and one cell line expressed a truncated RII transcript (de Jonge et al, 1997). The exact position of the truncation was not determined, but it was located within exon 4. However, the RII gene was screened for mutations in only four of the cell lines. In contrast, we did not find a truncated RNA transcript, but rather a mutation that would lead to a truncated receptor if translated.

From the present data it is not possible to deduce the succession of pathogenetic events; i.e. whether the mutation was preceded by silencing of the RII gene or vice versa. However, the type of mutation implies that this may not be an in vitro phenomenon, since occurrence of the G to T transversion in lung cancer mutational hotspots was previously shown to correlate with the formation of carcinogenic benzo[a]-pyrene metabolites found in cigarette smoke (Denissenko et al, 1996). Benzo[a]-pyrene induces specific mutations of the *p53* gene and, interestingly, a similar G to T transversion was also found in the *p53* gene of this particular SCLC cell line (Abrahamsen et al, unpublished results), supporting the assumption that the mutation of RII could have been a result of cigarette smoking. To our knowledge this kind of mutation of the RII gene has not been described previously.

In cancers, methylation of CpG islands in the promoter regions of various genes is one established mechanism for regulating transcriptional activity (Ferguson et al, 1995; Gonzalez-Zulaeta et al, 1995; Graff et al, 1995). The RII gene has been mapped to chromosome 3p22 (Mathew et al, 1994) and tumour suppressor genes associated with lung carcinogenesis have been suggested to be present on chromosome 3p since loss of heterozygosity at this chromosome occurs frequently in SCLC (Takenoshita et al, 1997). Furthermore, it has been shown that nearby loci to the location of the RII gene are hypermethylated in SCLC (Makos et al, 1995). However, we did not find any altered restriction pattern of the RII promoter. This is in agreement with a previous report where no hypermethylation was found of the RII promoter (de Jonge et al, 1997). We found two cell lines with an altered restriction pattern of the RII gene when digested with *Sma*I, but transcription of RII was not increased with 5-azaCdR treatment. Thus, the RII gene of these two cell lines must harbour an alteration at a *Sma*I site of unknown position, possibly in an intron, since no mutations in *Sma*I sites in the promoter or in the gene at exon 4 were found. The significance of the alteration in these two cell lines in relation to lack of RII expression remains uncertain. In gastric cancer cell lines genomic alterations of the RII gene have been described, both deletions and amplifications (Park et al, 1994). However, our observation is not quite similar, since only a specific combination of REs revealed a genomic alteration of the RII gene.

Our results indicate that alterations of the RII gene per se are not a major genetic basis of the malignant phenotype of SCLC, in contrast to gastrointestinal cancers (Markowitz et al, 1995). We did not find a high frequency of RII mutations in SCLC and we did not find an altered methylation pattern of the gene or promoter, which could account for the observed lack of RII expression. The cause of the silencing of the RII gene transcript is so far unknown, but may be due to alterations or defects in the transcriptional apparatus.

### ACKNOWLEDGEMENTS

The authors would like to thank Anna Chytil and Jette Røhmann for excellent technical assistance and for the photographic service of Thomas Carlslund. This study was supported by grants from the Danish Cancer Society, The Danish Cancer Research Foundation and the Astrid Thaysen Foundation.

### REFERENCES

Berendsen HH, De Leij L, De Vries EGE, Mesander G, Mulder NH, De Jong B, Buys CHCM, Postmus PE, Poppema S, Sluiter HJ and The HT (1988) Characterization of three small cell lung cancer cell lines established from one patient during longitudinal follow-up. *Cancer Res* **48**: 6891–6899

Carney DN, Gazdar AF, Bepler G, Guccion JG, MarangoS PJ, Moody TW, Zweig MH and Minna JD (1985) Establishment and identification of small cell lung cancer cell lines having classic and variant features. *Cancer Res* **45**: 2913–2923

Cui W, Kemp CJ, Duffie E, Balmain A and Akhurst RJ (1994) Lack of transforming growth factor-β1 expression in benign skin tumors of $p53^{null}$ mice is prognostic for a high risk of malignant conversion. *Cancer Res* **54**: 5831–5836

Cui W, Fowlis DJ, Bryson S, Duffie E, Ireland H, Balmain A and Akhurst RJ (1996) TGF-β1 inhibits the formation of benign skin tumors, but enhances progression to invasive spindle carcinomas in transgenic mice. *Cell* **86**: 531–542

Damstrup L, Rygaard K, Spang-Thomsen M and Poulsen HS (1993) Expression of the transforming growth factor β (TGFβ) receptors and TGF-β1, TGF-β2 and TGF-β3 in human small cell lung cancer cell lines. *Br J Cancer* **67**: 1015–1021

de Jonge RR, Garrigue-Antar L, Velluci VF and Reiss M (1997) Frequent inactivation of the transforming growth factor β type II receptor in small-cell lung carcinoma cells. *Oncology Res* **9**: 89–98

De Leil L, Postmus PE, Buys CHCM, Elema JD, Ramaekers F, Poppema S, Brouwer M, Van Der Veen AY, Mesander G and The TH (1985) Characterization of three new variant type cell lines derived from small cell carcinoma of the lung. *Cancer Res* **45**: 6024–6033

Denissenko MF, Pao A, Tang M-S and Pfeifer GP (1996) Preferential formation of benzoe[a]pyrene adducts at lung cancer mutational hotspots in *p53*. *Science* **274**: 430–432

Engelholm SA, Spang-Thomsen M, Vindeløv LL, Brünner N, Nielsen MH, Hirsch F and Hansen HH (1986) Comparison of characteristics of human small cell carcinoma of the lung in patients, in vitro and transplanted into nude mice. *Acta Pathol Microbiol Scand Sect A Pathol* **94**: 325–336

Ferguson AT, Lapidus RG, Baylin SB and Davidson NE (1995) Demethylation of the estrogen receptor gene in estrogen receptor-negative breast cancer cells can activate estrogen receptor expression. *Cancer Res* **55**: 2279–2283

Garrague-Antar L, Muñoz-Antonia T, Antonia SJ, Gesmonde J, Velluci VF and Reiss M (1995) Missense mutations of the transforming growth factor β type II receptor in human head and neck squamous carcinoma cells. *Cancer Res* **55**: 3982–3987

Geiser AG, Burmester JK, Webbink R, Roberts AB and Sporn MB (1992) Inhibition of growth by transforming growth factor-β following fusion of two nonresponsive human carcinoma cell lines. *J Biol Chem* **267**: 2588–2593

Gonzalez-Zulueta M, Bender CM, Yang AS, Nguyen T, Beart RW, van Tornout JM and Jones PJ (1995) Methylation of 5′CpG island of *p16*/*CDKN2* tumor suppressor gene in normal and transformed human tissues correlates with gene silencing. *Cancer Res* **55**: 4531–4535

Graff JR, Herman JG, Lapidus RG, Chopra H, Xu R, Jarnard PF, Isaacs WB, Pith PM, Davidson PM, Davidson NE and Baylin SB (1995) E-cadherin expression is silenced by DNA hypermethylation in human breast and prostate carcinomas. *Cancer Res* **55**: 5195–5199

Inagaki M, Moustakas A, Lin HY, Lodish HF and Carr BI (1993) Growth inhibition by transforming growth factor β (TGF-β) type 1 is restored in TGF-β-resistant hepatoma cells after expression of TGF-β receptor type II cDNA. *Proc Natl Acad Sci USA* **90**: 5359–5363

Kingsley DM (1994) The TGF-β superfamily: new members, new receptors, and new genetic tests of function in different organisms. *Genes Dev* **8**: 133–146

Liabakk N, Talbot I, Smith RA, Wilkinson K and Balkwill F (1996) Matrix metalloprotease 2 (MMP-2) and matrix metalloprotease 9 (MMP-9) type IV collagenases in colorectal cancers. *Cancer Res* **56**: 190–196

Lin HY, Wang X-F, Ng-Eaton E, Weinberg RA and Lodish HF (1992) Expression cloning of the TGF-β type II receptor, a functional transmembrane serine/threonine kinase. *Cell* **68**: 775–785

Lu S-L, Akiyami Y, Nagasaki H, Saiton K and Yuasa Y (1995) Mutations of the transforming growth factor-beta type II receptor gene and genomic instability in hereditary nonpolyposis colorectal cancer. *Biochem Biophys Res Commun* **216**: 452–457

Lu S-L, Zhang W-C, Akiyami Y, Nomizu T and Yuasa Y (1996) Genomic structure of the transforming growth factor β type II receptor gene and its mutations in hereditary nonpolyposis colorectal cancers. *Cancer Res* **56**: 4595–4598

Makos M, Nelkin BD, Lerman MI, Latif F, Zbar B and Baylin SB (1995) Distinct hypermethylation patterns occur at altered chromosome loci in human lung and colon cancer. *Proc Natl Acad Sci USA* **89**: 1929–1933

Markowitz S, Wang J, Myeroff L, Parsons R, Sun L, Lutterbaugh J, Fan RS, Zborowska E, Kinzler KW, Vogelstein B, Brattain M and Willson JKV (1995) Inactivation of the type II TGF-β receptor in colon cancer cells with microsatellite instability. *Science* **268**: 1336–1338

Mathew S, Murty VVVS, Cheifetz S, George D, Massagué J and Chaganti RSK (1994) Transforming growth factor receptor gene TGFβR2 maps to human chromosome band 3p22. *Genomics* **20**: 114–115

Mitsudomi T, Ogana T, Nishida A, Usaki T, Sugio K, Yasumo K, Sugimachi K and Gazdar AF (1996) Loss of heterozygosity at 3p in non-small cell lung cancer and its prognostic implications. *Clin Cancer Res* **2**: 1185–1188

Moses HL (1992) TGFβ regulation of epithelial cell proliferation. *Mol Reprod Dev* **32**: 179–184

Myeroff L, Parsons R, Kim S-J, Hedrik L, Rho KR, Orth K, Mathis M, Kinzler KW, Lutterbaugh J, Park K, Bang Y-J, Lee HY, Park J-G, Lynch HT, Roberts AB, Vogelstein B and Markowitz SD (1995) Transforming growth factor β receptor type II gene mutation common in colon and gastric but rare in endometrial cancers with microsatellite instability. *Cancer Res* **55**: 5545–5547

Nørgaard P, Damstrup L, Rygaard K, Spang-Thomsen M and Poulsen HS (1994) Growth suppression by transforming growth factor-β1 of human small cell lung cancer cell lines is associated to expression of the type II receptor. *Br J Cancer* **69**: 802–808

Nørgaard P, Hougaard S, Spang-Thomsen M and Poulsen HS (1995) Transforming growth factor β and cancer. *Cancer Treat Rev* **21**: 367–403

Nørgaard P, Spang-Thomsen M and Poulsen HS (1996) Expression and autoregulation of transforming growth factor β receptor mRNA in small-cell lung cancer cell lines. *Br J Cancer* **73**: 1037–1043

Park K, Kim S-J, Bang Y-J, Park J-G, Kim NK, Roberts AB and Sporn MB (1994) Genetic changes in the transforming growth factor β (TGF-β) type II receptor gene in human gastric cancer cells: correlation with sensitivity to growth inhibition by TGF-β. *Proc Natl Acad Sci USA* **91**: 8772–8776

Pettengill OS, Sorenson GD, Wurster-Hill D, Curphey TJ, Noll WW, Cate CC and Maurer LH (1980) Isolation and growth characteristics of continuous cell lines from small-cell carcinoma of the lung. *Cancer (Phila.)* **45**: 906–918

Pierce DF, Gorska AE, Chytil A, Meise KS, Page DL, Coffey RJ Jr and Moses HL (1995) Mammary tumor suppression by transforming growth factor β1 transgene expression. *Proc Natl Acad Sci USA* **92**: 4254–4258

Roberts AB and Sporn MB (1990) *The Transforming Growth Factor-βs. Handbook of Experimental Pharmacology. Peptide Growth Factors and Their Receptors*, Sporn MB and Roberts AB (eds), pp. 419–472. Springer Verlag: Heidelberg

Sambrook J, Fritsch EF and Maniatis T (1989) *Molecular Cloning*: *A Laboratory Manual*, 2nd edn. Cold Spring Harbor Laboratory: Cold Spring Harbor, NY

Sun L, Wu G, Willson JKV, Zborowska E, Yang J, Rajkarunanayake I, Wang J, Gentry LE, Wang X-F and Brattain MG (1994) Expression of transforming growth factor β type II receptor leads to reduced malignancy in human breast cancer MCF-7 cells. *J Biol Chem* **269**: 26449–26455

Takenoshita S, Hagiwara K, Gemma A, Nagashima M, Ryberg D, Lindstedt BA, Bennett WP, Haugen A and Harris CC (1997) Absence of mutations in the

© Cancer Research Campaign 1999

transforming growth factor-beta type II receptor in sporadic lung cancers with microsatellite instability and rare H-ras1 alleles. *Carcinogenesis* **18**: 1427–1429

Tani M, Takenoshita S, Kohno T, Hagiwara K, Nagamachi Y, Harris CC and Yokota JRA (1997) Infrequent mutations of the transforming growth factor beta type II receptor gene at chromosome 3p22 in human lung cancers with chromosome 3p deletions. *Carcinogenesis* **18**: 1119–1121

Todd S, Franklin WA, Varella-Garcia M, Kennedy T, Hillikwer CE, Hahner JrL, Anderson M, Wiest JS, Drabkin HA and Gemmill RM (1997) Homozygous deletions of human chromosome 3p in lung tumors. *Cancer Res* **57**: 1344–1352

Vincent F, Nagashima M, Takenoshita S, Khan MA, Gemma A, Hagiwara K and Bennett WP (1997) Mutation analysis of the transforming growth factor-beta type II receptor in human cell lines resistant to growth inhibition by transforming growth factor-beta. *Oncogene* **15**: 117–122

Wang J, Sun L, Myeroff L, Wang X, Gentry LE, Yang J, Liang J, Zborowska E, Markowitz S, Willson JK and Brattain MG (1995) Demonstration that mutation of the type II transforming growth factor β receptor inactivates its tumor suppressor activity in replication error-positive colon carcinoma cells. *J Biol Chem* **270**: 22044–22049

Wrana JL, Attisano L, Wieser F, Ventura F and Massagué J (1994) Mechanism of activation of the TGF-β receptor. *Nature* **370**: 341–347

© Cancer Research Campaign 1999 
